# On the stress-strain alignment in premixed turbulent flames

**DOI:** 10.1038/s41598-019-41599-y

**Published:** 2019-03-25

**Authors:** Umair Ahmed, Nilanjan Chakraborty, Markus Klein

**Affiliations:** 1 School of Engineering, Newcastle University, Newcastle Upon-Tyne, NE1 7RU UK; 2 Bundeswehr University Munich, Department of Aerospace Engineering, Werner Heisenberg Weg 39, D-85577 Neubiberg, Germany

## Abstract

The interaction of large and small scale structures is fundamental to the energy cascade in turbulent flows. The correct representation of this interaction becomes important in the context of large eddy simulation (LES), where the response of small-scale structures to the resolved quantities, or large-scale structures, must be parametrised. This challenging task becomes more demanding when LES of premixed flames are considered, as heat release affects the interaction of turbulence and chemistry occurring at the unresolved scales. In this work, the influence of sub-grid scale (SGS) stresses on the kinetic energy budget of the resolved velocity field in turbulent premixed flames is investigated. In this spirit, the alignment between the SGS stresses and the resolved strain rate has been analysed by interrogating a direct numerical simulation (DNS) database of statistically planar premixed flames subjected to forced isotropic turbulence. It has been found that the alignment between the eigenvectors of the SGS stresses and the resolved strain rate changes across the flame brush and this change is dependent on the level of turbulence experienced by the flame. The influence of different turbulence intensities and different filter widths along with the implications of this misalignment on the SGS modelling are discussed in detail in the paper.

## Introduction

The interplay of large and small scale structures is central to the energy transfer in turbulent flows. Turbulence is inherently three-dimensional and spans a wide range of length scales, ranging from the large energy containing scales to the small viscous dissipation scales. It is important to characterise the features of the three-dimensional structure of interactions amongst these different scales^1^. An accurate representation of this interplay becomes important in the context of large eddy simulation (LES), where the influences of small-scale structures on the resolved turbulent fields, or large-scale structures, need to be appropriately accounted for. This requirement becomes more stringent in turbulent premixed flames, as there is a strong coupling between turbulence and chemical reactions occurring at the unresolved scales. In the case of non-reacting flows several studies^1–3^ have focused on the interplay between large and small scales and their influence on the closure of the sub-grid scale (SGS) stress tensor in the context of LES. In this work we focus on the aforementioned behaviour in premixed turbulent flames. In LES, the small scales are represented by the SGS stress tensor, defined as:

1 $$\bar{\rho }{\tilde{\tau }}_{ij}^{SGS}=\overline{\rho {u}_{i}{u}_{j}}-\bar{\rho }{\tilde{u}}_{i}{\tilde{u}}_{j},$$

where the filtering operator is defined as $\overline{Q({\boldsymbol{x}})}=\int \,Q({\boldsymbol{x}}-{\boldsymbol{r}}){\boldsymbol{G}}({\boldsymbol{r}}){\boldsymbol{dr}}$, where *G*(***r***) is the filter kernel and $\tilde{Q}=\overline{\rho Q}/\bar{\rho }$ denotes the Favre filtered value of a general variable *Q*. One of the most important features of ${\tilde{\tau }}_{ij}^{SGS}$ is how it influences the kinetic energy budget of the resolved velocity field^1,2^. The dominant effect is through the resolved (SGS) kinetic energy dissipation (production) **Γ** which is a consequence of the interaction between sub-grid and resolved scales defined as:

2 $${\boldsymbol{\Gamma }}=-\,{\tilde{\tau }}_{ij}^{SGS}{\tilde{S}}_{ij},$$

where ${\tilde{S}}_{ij}$ is the Favre filtered strain rate tensor $({\tilde{S}}_{ij}=\mathrm{0.5(}\partial {\tilde{u}}_{i}/\partial {x}_{j}+\partial {\tilde{u}}_{j}/\partial {x}_{i}))$. The resolved-scale kinetic energy dissipation can be written as ${\boldsymbol{\Gamma }}={\rm{trace}}\,(-\,{\tilde{{\boldsymbol{\tau }}}}^{SGS}\cdot \tilde{{\boldsymbol{S}}})$, where both ${\tilde{\tau }}_{ij}^{SGS}$ and ${\tilde{S}}_{ij}$ are symmetric and real. The above discussion indicates that the relative alignment between the SGS stress and resolved strain rate plays a key role in determining the dissipation rate of the resolved kinetic energy. Thus, the relative alignment between the SGS stress and the resolved strain rate is expected to play a pivotal role for both turbulence and combustion modelling in premixed flames.

The tensors ${\tilde{\tau }}_{ij}^{SGS}$ and ${\tilde{S}}_{ij}$ can be decomposed into base eigenvectors using eigendecomposition as:

3 $${\tilde{S}}_{ij}={\alpha }_{s}{{\boldsymbol{\alpha }}}_{s}{{\boldsymbol{\alpha }}}_{s}^{T}+{\beta }_{s}{{\boldsymbol{\beta }}}_{s}{{\boldsymbol{\beta }}}_{s}^{T}+{\gamma }_{s}{{\boldsymbol{\gamma }}}_{s}{{\boldsymbol{\gamma }}}_{s}^{T},$$

4 $$-{\tilde{\tau }}_{ij}^{SGS}={\alpha }_{-\tau }{{\boldsymbol{\alpha }}}_{-\tau }{{\boldsymbol{\alpha }}}_{-\tau }^{T}+{\beta }_{-\tau }{{\boldsymbol{\beta }}}_{-\tau }{{\boldsymbol{\beta }}}_{-\tau }^{T}+{\gamma }_{-\tau }{{\boldsymbol{\gamma }}}_{-\tau }{{\boldsymbol{\gamma }}}_{-\tau }^{T},$$

where *α*, *β* and *γ* are the eigenvalues and ***α***, ***β*** and ***γ*** are the respective eigenvectors; the subscripts *s* and −*τ* represent the respective eigenvalues and eigenvectors of the resolved strain rate and the negative of SGS stress tensors, respectively, and the transposed vector is represented by the superscript *T*. The eigenvalues are ordered as *α* > *β* > *γ*, and the corresponding eigenvectors ***α***, ***β*** and ***γ*** are labelled as the extensive, intermediate and compressive eigenvectors, respectively. Note that the negative sign has been included in the SGS stresses in Eq. 4 as in the case of eddy viscosity models $$-{\tilde{\tau }}_{ij}^{SGS}+({\delta }_{ij}\mathrm{/3)}{\tilde{\tau }}_{kk}^{SGS}=2{\nu }_{t}{\tilde{S}}_{ij}-\mathrm{(2}{\nu }_{t}\mathrm{/3}{\delta }_{ij})(\partial {\tilde{u}}_{k}/\partial {x}_{k})$$, where *ν*_*t*_ is the kinematic eddy viscosity (*ν*_*t*_ = (*C*_*s*_Δ)^2^(2$${\tilde{S}}_{ij}$$$${\tilde{S}}_{ij}$$)^1/2^, where *C*_*s*_ is the model constant and Δ is the filter width). Different variants of closures for $$-{\tilde{\tau }}_{ij}^{SGS}$$ exist in the literature for premixed combustion and further details can be found in Klein *et al*.^4^. The resolved kinetic energy dissipation can be expressed as^5^:

5 $$\begin{array}{rcl}{\boldsymbol{\Gamma }} & = & {\alpha }_{-\tau }{\alpha }_{s}{({{\boldsymbol{\alpha }}}_{-\tau }\cdot {{\boldsymbol{\alpha }}}_{s})}^{2}+{\alpha }_{-\tau }{\beta }_{s}{({{\boldsymbol{\alpha }}}_{-\tau }\cdot {{\boldsymbol{\beta }}}_{s})}^{2}\\  &  & +\,{\alpha }_{-\tau }{\gamma }_{s}{({{\boldsymbol{\alpha }}}_{-\tau }\cdot {{\boldsymbol{\gamma }}}_{s})}^{2}+{\beta }_{-\tau }{\alpha }_{s}{({{\boldsymbol{\beta }}}_{-\tau }\cdot {{\boldsymbol{\alpha }}}_{s})}^{2}\\  &  & +\,{\beta }_{-\tau }{\beta }_{s}{({{\boldsymbol{\beta }}}_{-\tau }\cdot {{\boldsymbol{\beta }}}_{s})}^{2}+{\beta }_{-\tau }{\gamma }_{s}{({{\boldsymbol{\beta }}}_{-\tau }\cdot {{\boldsymbol{\gamma }}}_{s})}^{2}\\  &  & +\,{\gamma }_{-\tau }{\alpha }_{s}{({{\boldsymbol{\gamma }}}_{-\tau }\cdot {{\boldsymbol{\alpha }}}_{s})}^{2}+{\gamma }_{-\tau }{\beta }_{s}{({{\boldsymbol{\gamma }}}_{-\tau }\cdot {{\boldsymbol{\beta }}}_{s})}^{2}\\  &  & +\,{\gamma }_{-\tau }{\gamma }_{s}{({{\boldsymbol{\gamma }}}_{-\tau }\cdot {{\boldsymbol{\gamma }}}_{s})}^{2},\end{array}$$

where (***a***⋅***b***) = cos*θ* and *θ* is the angle between the vectors ***a*** and ***b***. Thus, the resolved kinetic energy dissipation is determined by the joint statistics of geometric alignments of the SGS stress and strain rate tensors and their respective eigenvalues.

**Figure 1:**
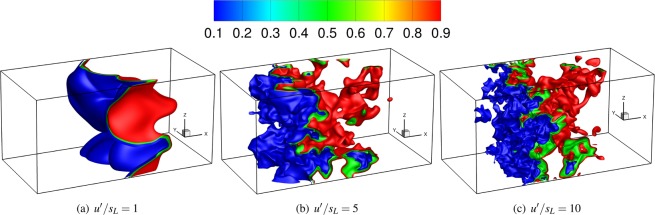
Iso-surfaces of the progress variable for all the cases investigated.

It has been shown in earlier studies on non-reacting flows ranging from isotropic turbulence^3^ to shear flows^5^ that the eigenvectors of ${\tilde{S}}_{ij}$ and $-{\tilde{\tau }}_{ij}^{SGS}$ are not aligned. The influence of heat release on the alignment of ${\tilde{S}}_{ij}$ and $-{\tilde{\tau }}_{ij}^{SGS}$ has not been analysed yet. In this work, we analyse the aforementioned alignment of the SGS stresses and the resolved strain rate tensor by interrogating a direct numerical simulation (DNS) database of statistically planar flames subjected to forced isotropic turbulence as listed in Table 1, where *Da* = *l*_*t*_*s*_*L*_/*u*′*δ*_*th*_, *Ka* = (*u*′/*s*_*L*_)^1.5^(*l*_*t*_/*δ*_*th*_)^−0.5^ and *Re*_*t*_ are the Damköhler, Karlovitz and turbulent Reynolds number based on unburned gas properties and integral length scale, respectively (with *l*_*t*_ being the integral length scale, *u*′ the root mean squared velocity fluctuation, *s*_*L*_ the unstrained laminar flame speed and *δ*_*th*_ the thermal flame thickness defined as *δ*_*th*_ = (*T*_*ad*_ − *T*_*R*_)/max|▿*T*|_*L*_; where *T*, *T*_*ad*_ and *T*_*R*_ are the instantaneous temperature, the adiabatic flame temperature and the reactant gas temperature respectively. The subscript *L* refers to the unstrained laminar flame quantities). Three simulations of unity Lewis number with different turbulence intensities, *Ka*, *Da* and *Re*_*t*_, have been performed. A rectangular domain as shown in Fig. 1 of size 140.5*δ*_*z*_ × 70.18*δ*_*z*_ × 70.18*δ*_*z*_ has been considered (where *δ*_*z*_ = *α*_*T*_/*s*_*L*_ is the Zeldovich flame thickness, *α*_*T*_ is the thermal diffusivity of the unburnt mixture). Figure 1 shows the instantaneous realisation of the progress variable *c* (the progress variable in this analysis is defined using the normalised fuel mass fraction (i.e. $c=({Y}_{{F}_{R}}-{Y}_{F})/({Y}_{{F}_{R}}-{Y}_{{Y}_{{F}_{P}}})$ where *Y*_*F*_ is the fuel mass fraction and subscripts *R* and *P* refer to values in unburned reactants and fully burned products, respectively) for the three flames investigated.

**Table 1:** Turbulence parameters for different cases investigated.

	*u*′/*s*_*L*_	*Ka*	*Da*	${\boldsymbol{R}}{{\boldsymbol{e}}}_{{\boldsymbol{t}}}$
Case-A	1.0	0.58	3.0	17.8
Case-B	5.0	6.5	0.6	89.1
Case-C	10.0	18.3	0.3	178.1

## Results and Discussion

**Figure 2:**
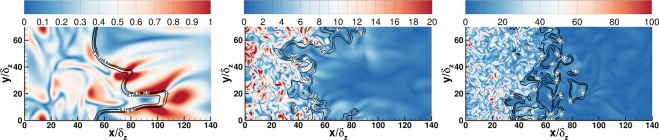
Instantaneous slices of vorticity magnitude ($\sqrt{{\omega }_{i}{\omega }_{i}}\times {\delta }_{th}/{s}_{L}$) for case-A (left), case-B (middle) and case-C (right). Black lines represent the iso-surfces of the progress variable.

Figure 2 shows the contours of the progress variable superimposed on the vorticity magnitude field, where vorticity is defined as *ω*_*i*_ = *ε*_*ijk*_∂*u*_*k*_/∂*x*_*j*_. It can be seen in case-A that the turbulence only wrinkles the flame and does not enter the flame structure, whereas in the case of higher turbulence intensity the turbulence can enter the flame structure and can distort the preheat zone of the flame. Furthermore, the vorticity is amplified across the flame structure in case-A, whereas the vorticity is attenuated by the flame in higher turbulence intensity cases (i.e. case-B and case-C). This behaviour of the flame structure is consistent with previous studies^6,7^ involving flames at high *Ka* and demonstrates the differences in turbulence-flame interaction with varying turbulence intensity. These variations have implications on the behaviour of stress-strain alignment and are discussed in the following subsections.

### Sub-grid stresses in the principal strain rate coordinate system

In order to investigate the orientation of the principal axes of the normalised sub-grid scale stress tensor (${\tilde{{\rm{\Pi }}}}_{ij}=-\,{\tilde{\tau }}_{ij}^{SGS}/{\tilde{k}}_{SGS}$, where *k*_*SGS*_ is the sub-grid turbulent kinetic energy), we first consider it in the principal coordinate system of the local resolved strain rate tensor. The SGS stress tensor can be expressed in the principal coordinate system of the strain rate tensor via a coordinate transform $\hat{{\rm{\Pi }}}={\tilde{{\boldsymbol{E}}}}^{T}\tilde{{\boldsymbol{\Pi }}}\tilde{{\boldsymbol{E}}}$, where ${\tilde{{\boldsymbol{E}}}}^{T}$ and $\tilde{{\boldsymbol{E}}}$ are the orthogonal matrices with rows and columns ***α***_*s*_, ***β***_*s*_ and ***γ***_*s*_ respectively.

**Figure 3:**
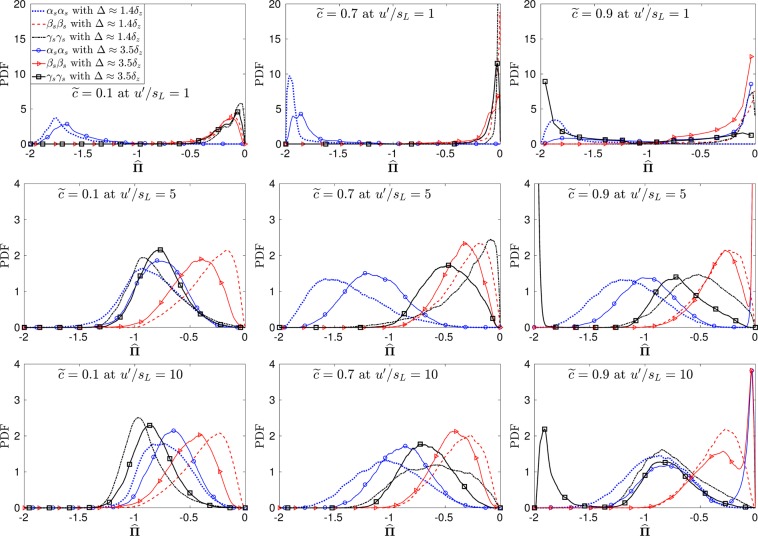
Pdfs for the diagonal components of $\hat{{\boldsymbol{\Pi }}}$ at Δ ≈ 1.4*δ*_*z*_ and Δ ≈ 3.5*δ*_*z*_.

Note that $\hat{{\boldsymbol{\Pi }}}$ is a symmetric tensor, hence ${\hat{{\rm{\Pi }}}}_{{\gamma }_{s}{\alpha }_{s}}={\hat{{\rm{\Pi }}}}_{{\alpha }_{s}{\gamma }_{s}}$, ${\hat{{\rm{\Pi }}}}_{{\alpha }_{s}{\beta }_{s}}={\hat{{\rm{\Pi }}}}_{{\beta }_{s}{\alpha }_{s}}$ and ${\hat{{\rm{\Pi }}}}_{{\beta }_{s}{\gamma }_{s}}={\hat{{\rm{\Pi }}}}_{{\gamma }_{s}{\beta }_{s}}$. It is usually argued that of the six independent components of $\hat{{\boldsymbol{\Pi }}}$, the three diagonal components are of primary importance as they directly interact with the strain rate eigenvalues and lead to the magnification of the velocity gradients. To confirm the significance of the three diagonal components of $\hat{{\boldsymbol{\Pi }}}$ in premixed flames, the probability density functions (PDFs) of ${\hat{{\rm{\Pi }}}}_{ii}$ have been plotted in Fig. 3. In case-A, for Δ ≈ 1.4*δ*_*Z*_, ${\hat{{\rm{\Pi }}}}_{{\alpha }_{s}{\alpha }_{s}}$ has the highest probability for non-zero SGS stresses across the whole flame brush (i.e. for all the values of ${\hat{{\rm{\Pi }}}}_{{\alpha }_{s}{\alpha }_{s}}$ shown in Fig. 3), whereas this is not the case for higher turbulence intensity flames (cases B and C). The high values of ${\hat{{\rm{\Pi }}}}_{{\alpha }_{s}{\alpha }_{s}}$ in case-A exist due to the effects of heat release in the flow field which lead to the dominance of ***α***_*s*_. In case-B, for Δ ≈ 1.4*δ*_*Z*_, there is a competition between ***α***_*s*_ and ***γ***_*s*_, hence there is an equal probability of finding non-zero values of ${\hat{{\rm{\Pi }}}}_{{\alpha }_{s}{\alpha }_{s}}$ and ${\hat{{\rm{\Pi }}}}_{{\gamma }_{s}{\gamma }_{s}}$ at $\tilde{c}=0.1$. This trend changes towards the region of heat release (at $\tilde{c}=0.7$) and at $\tilde{c}=0.9$ where ***α***_*s*_ dominates and consequently high probabilities for non-zero values for ${\hat{{\rm{\Pi }}}}_{{\alpha }_{s}{\alpha }_{s}}$ exist. A clear dominance of ***γ***_*s*_ can be seen in case-C, for Δ ≈ 1.4*δ*_*Z*_, which leads to high probability for non-zero values of ${\hat{{\rm{\Pi }}}}_{{\gamma }_{s}{\gamma }_{s}}$ at $\tilde{c}=0.1$. In the region of heat release (at $\tilde{c}=0.7$) in case-C the most extensive eigenvector of the strain rate dominates and leads to high non-zero values of ${\hat{{\rm{\Pi }}}}_{{\alpha }_{s}{\alpha }_{s}}$. At $\tilde{c}=0.9$ there is a competition between ***α***_***s***_ and ***γ***_***s***_ as the influence of dilatation starts to decrease in this region of the flame. In the case when a larger filter width (Δ ≈ 3.5*δ*_*z*_) is used for all flames the probability of non-zero values of ${\hat{{\rm{\Pi }}}}_{{\gamma }_{s}{\gamma }_{s}}$ increases at $\tilde{c}=0.9$, while the probabilities of obtaining non-zero values for $\hat{{\boldsymbol{\Pi }}}$_*ii*_ in the rest of the flame are not affected by this change.

**Figure 4:**
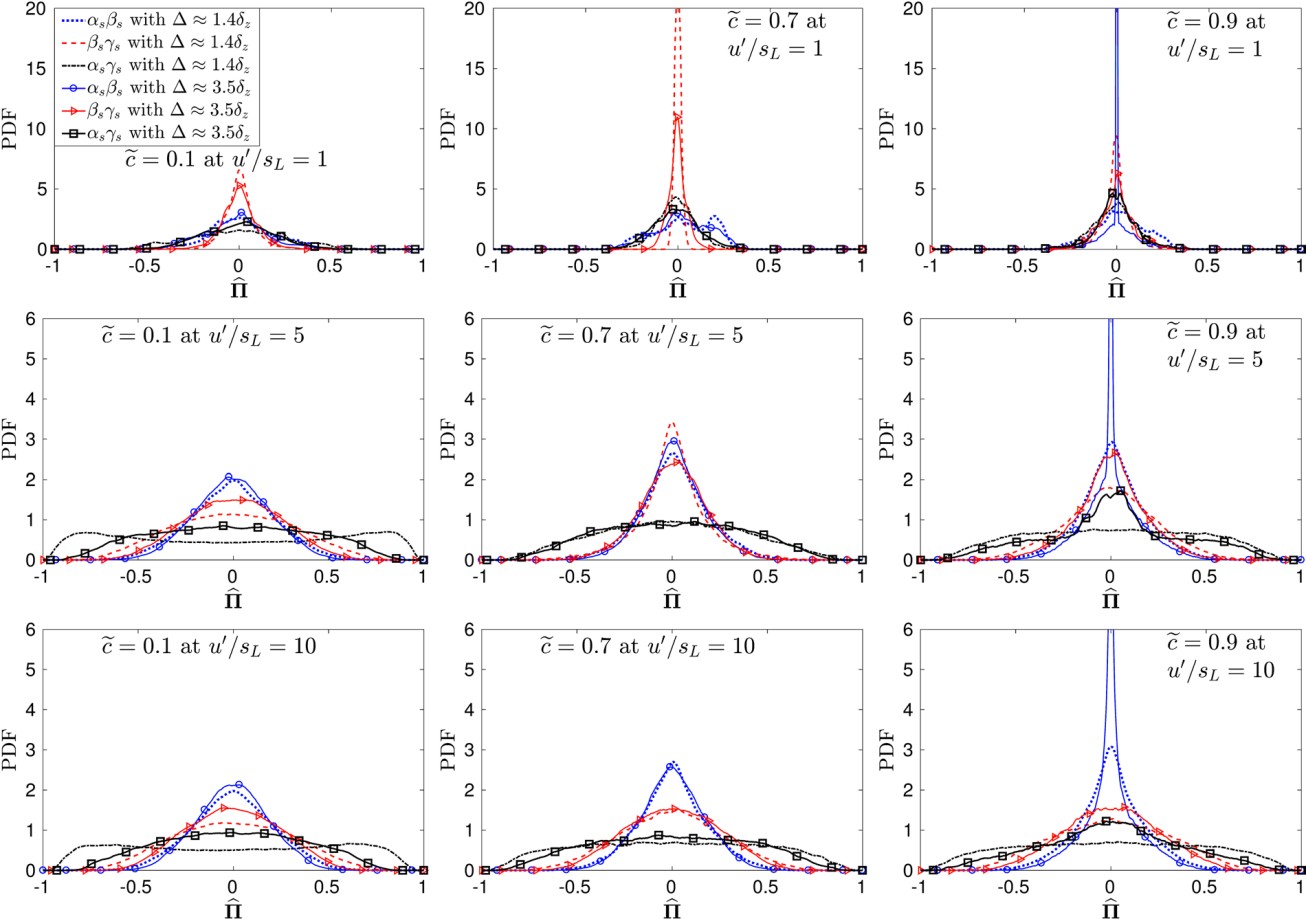
Pdfs for the off-diagonal components of $\hat{{\boldsymbol{\Pi }}}$ at Δ ≈ 1.4*δ*_*z*_ and Δ ≈ 3.5*δ*_*z*_.

**Figure 5:**
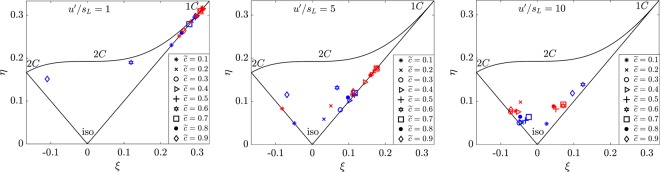
Lumley triangle on the plane of the invariants *ξ* and *η* of the transformed sub-grid scale stress anisotropy tensor. 1*C*, 2*C* and iso mean one component limit, two component limit and isotropic respectively. All of the sub-grid stresses have been calculated at Δ ≈ 1.4*δ*_*z*_ (coloured in red) and Δ ≈ 3.5*δ*_*z*_ (coloured in blue).

The PDFs for off-diagonal terms for $\hat{{\boldsymbol{\Pi }}}$ are presented in Fig. 4. The contributions of the off-diagonal terms for $\hat{{\boldsymbol{\Pi }}}$ are negligible in case-A for all the filter widths investigated. In cases B and C, the contribution from the off-diagonal terms increases across the flame brush and is insensitive to the choice of the filter width used. Figure 5 shows the behaviour of $\hat{{\boldsymbol{\Pi }}}$_*ij*_ on the Lumley triangle, where *η* and *ξ* represent the second and third invariants of the normalised anisotropy tensor for $\hat{{\boldsymbol{\Pi }}}$_*ij*_ defined as:

6 $$6{\eta }^{2}={b}_{ij}{b}_{ji},\,{\rm{and}}\,6{\xi }^{3}={b}_{ij}{b}_{jk}{b}_{ki},$$

where *b*_*ij*_ is defined as ${b}_{ij}={\hat{{\rm{\Pi }}}}_{ij}/{\hat{{\rm{\Pi }}}}_{ii}-\mathrm{1/3}{\delta }_{ij}$^8^. In case-A ${\hat{{\rm{\Pi }}}}_{ij}$ is highly anisotropic due to the increased contributions of ***α***_*s*_ terms in $\hat{{\boldsymbol{\Pi }}}$ which consequently leads to a one component limit behaviour. In the case of higher turbulence intensity (cases B and C) the level of anisotropy decreases with increasing turbulence intensity due to decreased contributions of the ***α***_*s*_ terms, thus leading to high levels of isotropy of $\hat{{\boldsymbol{\Pi }}}$_*ij*_. In flames with a combination of small *u*′/*s*_*L*_ and high *Da* (i.e. case-A), the dilatation (▿.***u***) almost equals ***α***_*s*_ and consequently the vorticity does not align with ***α***_*s*_^6^. Under these circumstances, the viscous dissipation (${\tilde{\varepsilon }}_{ij}$) reduces due to the lack of alignment between the vorticity and ***α***_*s*_ (as ${\tilde{\varepsilon }}_{ij}\sim \overline{\mu {\omega }_{i}{\omega }_{j}}/\bar{\rho }-\bar{\mu }/\bar{\rho }({\tilde{\omega }}_{i}{\tilde{\omega }}_{j})$); whereas the SGS stress generation due to pressure-strain correlation ($\overline{p(\partial {u}_{i}/\partial {x}_{j}+\partial {u}_{j}/\partial {x}_{i}})-\bar{p}(\partial {\tilde{u}}_{i}/\partial {x}_{j}+\partial {\tilde{u}}_{j}/\partial {x}_{i})$) remains strong in the direction of ***α***_*s*_. This results in a situation where the SGS stress tensor has only one significant component, which exists in the direction of ***α***_*s*_ and this is reflected in the one component like behaviour for case-A in Fig. 5. Since the SGS stresses and the resolved strain rate have a tendency to change alignment across the flame brush under different turbulence conditions, the relative alignment between the two eigensystems is investigated in the following subsection.

### Stress-strain alignment

**Figure 6:**
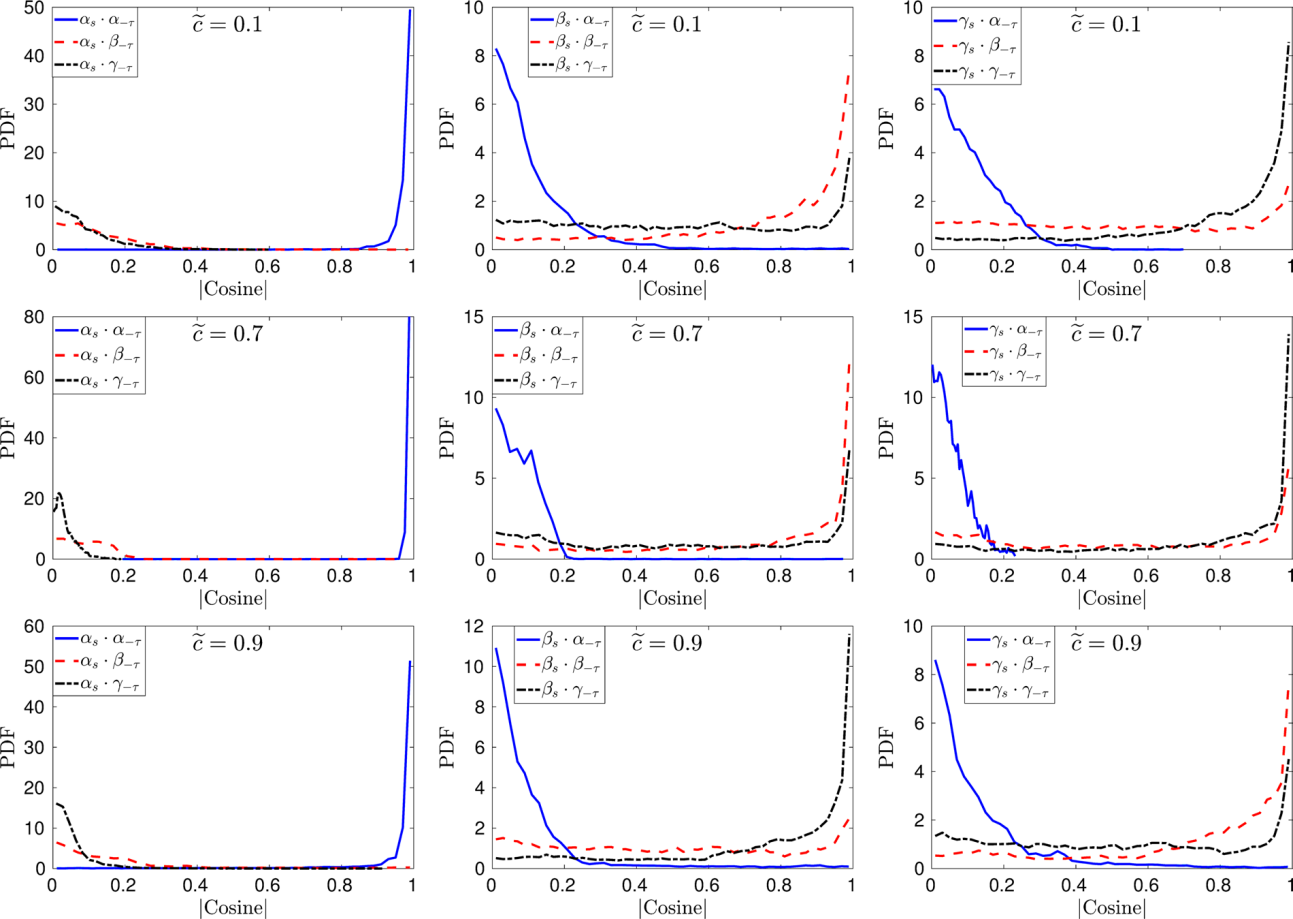
Pdfs of the direction cosines between the eigenvectors of ${\tilde{S}}_{ij}$ and $-{\tilde{\tau }}_{ij}^{SGS}$ at different iso-surfaces of the filtered progress variable for case-A (*u*′/*s*_*L*_ = 1) at Δ ≈ 1.4*δ*_*z*_.

**Figure 7:**
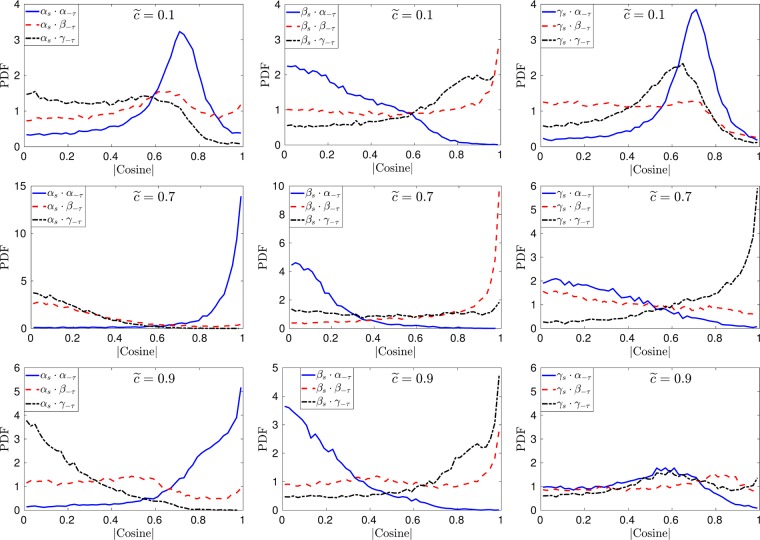
Pdfs of the direction cosines between the eigenvectors of ${\tilde{S}}_{ij}$ and $-{\tilde{\tau }}_{ij}^{SGS}$ at different iso-surfaces of the filtered progress variable for *u*′/*s*_*L*_ = 5 case at Δ ≈ 1.4*δ*_*z*_.

**Figure 8:**
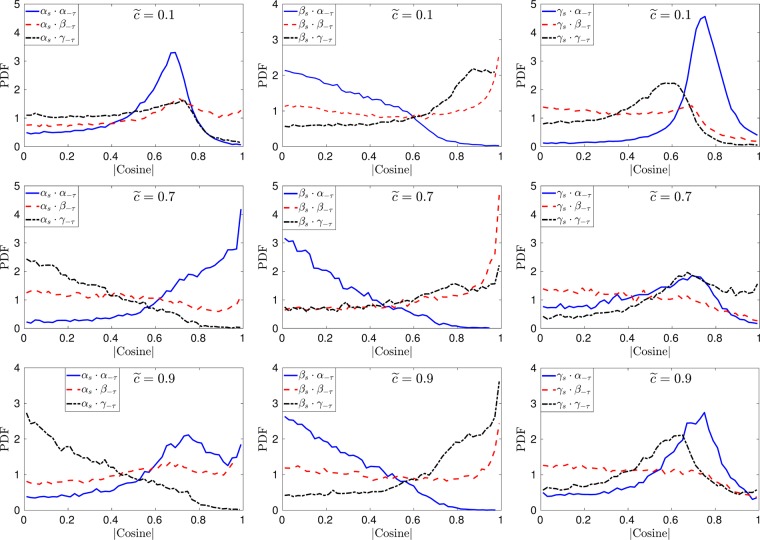
Pdfs of the direction cosines between the eigenvectors of ${\tilde{S}}_{ij}$ and $-{\tilde{\tau }}_{ij}^{SGS}$ at different iso-surfaces of the filtered progress variable for *u*′/*s*_*L*_ = 10 case at Δ ≈ 1.4*δ*_*z*_.

**Figure 9:**
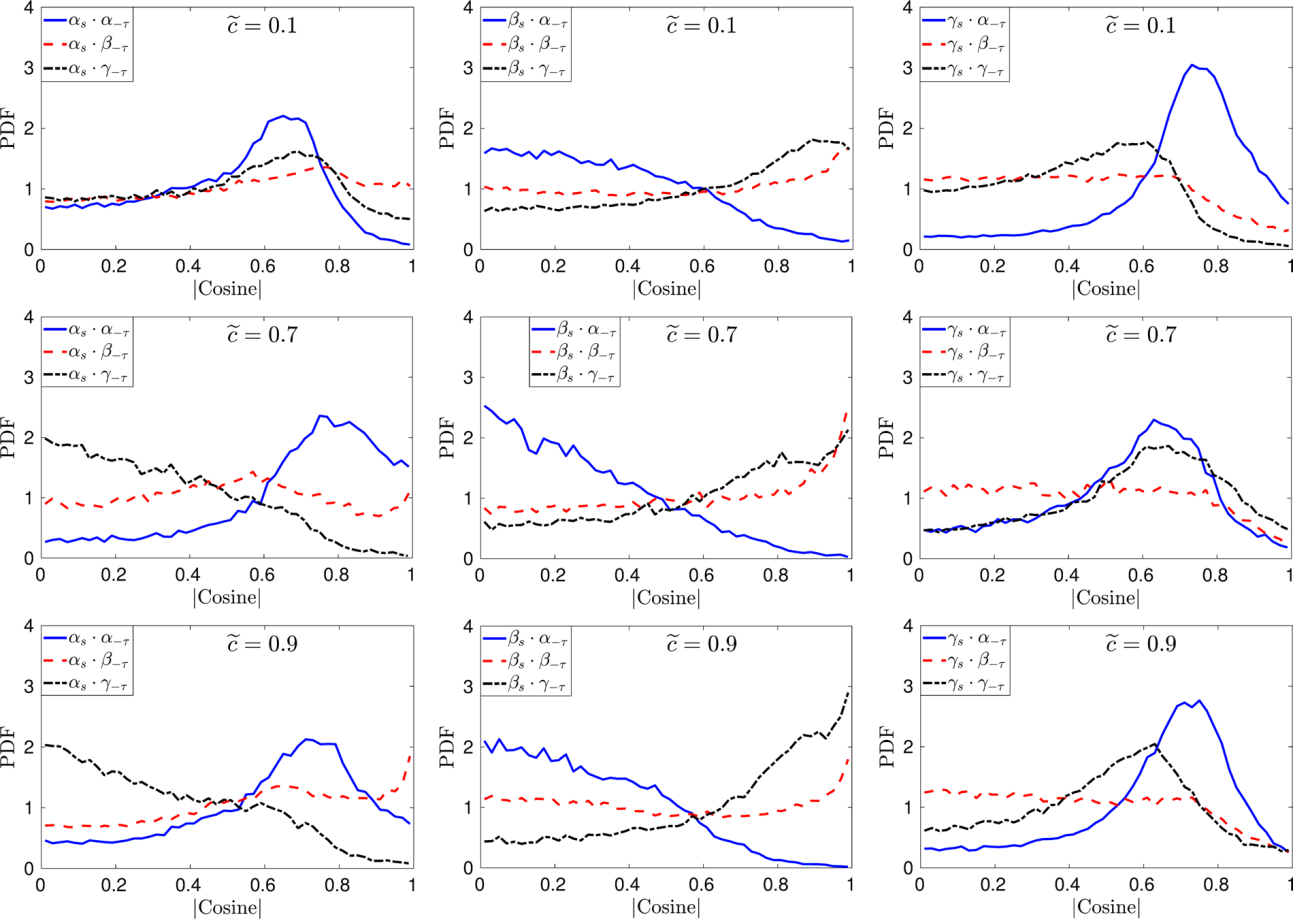
Pdfs of the direction cosines between the eigenvectors of ${\tilde{S}}_{ij}$ and $-{\tilde{\tau }}_{ij}^{SGS}$ at different iso-surfaces of the filtered progress variable for *u*′/*s*_*L*_ = 10 case at Δ ≈ 3.5*δ*_*z*_.

The alignment between the eigenvectors of ${\tilde{S}}_{ij}$ and $-{\tilde{\tau }}_{ij}^{SGS}$ has been investigated further by interrogating the PDFs of the direction cosine between the two eigensystems as plotted in Figs 6–9. It can be noticed from Fig. 6 that in case-A (*u*′/*s*_*L*_ = 1) the eigenvectors for $-{\tilde{\tau }}_{ij}^{SGS}$ and ${\tilde{S}}_{ij}$ are completely aligned for $\tilde{c}=0.1,\,\tilde{c}=0.7$ and $\tilde{c}=0.9$ (this is consistent for all the iso-surfaces of the flame and is not shown here), this is contrary to the earlier findings for non-reacting isotropic turbulence by Horiuti *et al*.^3^. The perfect alignment of the eigenvectors for the two tensors can be explained by the fact that in the case of reacting flows with heat release the effects of dilatation play an important role as shown in many previous studies involving scalar gradient alignment with the principal directions of the strain rate^9,10^. In the case of high *Da* flames the dilatation effects are strong which cause the SGS stresses to align perfectly with the strain rate and this is discussed in detail later on in this paper. The alignment of the two eigensystems changes at the trailing edge of the flame ($\tilde{c}=0.9$) and ***α***_*s*_ remains aligned with ***α***_−*τ*_ but the other two eigenvectors change alignment and rotate by 90°, which results in the alignment of ***β***_*s*_ with ***γ***_−*τ*_ and ***γ***_***s***_ with ***β***_−*τ*_.

In the case of premixed combustion, the relative alignment of the eigenvectors for ${\tilde{S}}_{ij}$ and $-{\tilde{\tau }}_{ij}^{SGS}$ is influenced by the competition between the thermochemical and fluid dynamic processes. This implies that the chemical reactions releasing heat cause dilatation and flame normal acceleration which competes with the local turbulent fluid dynamics processes. In an unstrained laminar premixed planar flame, the flame normal acceleration and dilatation scale as *τs*_*L*_/*δ*_*th*_. As the dilatation is induced by the chemical reactions, the inverse time scale related to dilatation (*τs*_*L*_/*δ*_*th*_) can be represented as the chemical strain rate, which scales as *τDa* when normalised using the integral time scale^9^. This scaling implies that the chemical strain rate, which acts in the flame normal direction, dominates over turbulent straining in high *Da* flames (case-A); thus causing the two eigensystems of the SGS stresses and the strain rate to become completely aligned throughout the flame structure. In the case of low *Da* flames the turbulent strain rate is stronger than the chemical strain rate and thus it is expected to have a non-reacting turbulence like behaviour. Under these conditions, the strain rate induced by flame normal acceleration is weaker than turbulent straining as demonstrated in previous analyses^9,11^. It is well known that the strain rate effects in premixed combustion are dependent on the size of the turbulence eddies^12^; which implies that the length scales of eddies play an important role in premixed turbulent combustion and consequently *Ka* dependence for this interaction becomes important.

The results for higher turbulence intensity cases B and C (*u*′/*s*_*L*_ = 5 and *u*′/*s*_*L*_ = 10) are presented in Figs 7 and 8 at different iso-surfaces of the filtered progress variable. Qualitatively the results for case-B and case-C are similar and the relative alignment between the respective eigenvectors of the resolved strain rate and the SGS stresses changes across the flame brush. At $\tilde{c}=0.1$, the extensive and compressive eigenvectors of ${\tilde{S}}_{ij}$ and $-{\tilde{\tau }}_{ij}^{SGS}$ are at approximately 45° to each other and the intermediate eigenvectors of the two tensors (i.e. ${\tilde{S}}_{ij}$ and $-{\tilde{\tau }}_{ij}^{SGS}$) have a high probability of collinear alignment. In the region of heat release ($\tilde{c}=0.7$), the intermediate eigenvectors remain aligned, while ***α***_*s*_ aligns with ***α***_−*τ*_ and ***γ***_*s*_ aligns with ***γ***_−*τ*_. The change in alignment occurs due to the fact that turbulence enters the flame structure in flames with *Ka* > 1 conditions and alters the strain rate behaviour towards that of the non-reacting turbulence. Similar behaviour has been noticed in several previous numerical and experimental studies for scalar turbulence interaction^9,13^. Similar to case-A, the alignment changes again on the product side of the flame in case-B and case-C. Note that in all cases at $\tilde{c}=0.9$ ***β***_*s*_ aligns with ***γ***_−*τ*_ which is different to the non-reacting turbulence which implies that the relative alignment between the two eigensystems is affected by the viscous action as a result of increased kinematic viscosity in the burned gas region.

The influence of filter width on the alignment between the eigenvectors of the SGS stress and the resolved strain rate tensor for case-C can be seen by comparing Figs 8 and 9. The alignment trends of the two eigensystems are slightly affected for case-B and case-C by the choice of the filter width used. Especially in the regions of heat release ($\tilde{c}\ge 0.7$) the alignment of the extensive and compressive eigenvectors is altered by increasing the filter width, whereas the alignment trends for lower turbulence intensity flames (case-A) are not affected by the change in filter width (not shown here). These results show that the SGS stresses are likely to change alignment with the resolved part of the strain rate in LES of turbulent reacting flows. The nature of this alignment is qualitatively different from non-reacting flows depending on Damköhler and Karlovitz numbers of the flame.

## Summary and Conclusions

The relative alignment of the negative of the sub-grid scale (SGS) stress $-{\tilde{\tau }}_{ij}^{SGS}$ and resolved strain rate ${\tilde{S}}_{ij}$ eigenvectors has been investigated for premixed turbulent flames. Three flames subjected to forced isotropic turbulence at different intensities have been interrogated to identify the effects of heat release on stress-strain alignment. The data are analysed in the context of large eddy simulation (LES) and it is found that the relative alignment of $-{\tilde{\tau }}_{ij}^{SGS}$ and ${\tilde{S}}_{ij}$ is altered in the presence of heat release. In the case of high Damköhler number (*Da*) flames the eigenvectors for the SGS stress and strain rate are completely aligned throughout the flame structure, whereas in the case of low *Da* and high Karlovitz number (*Ka*) flames the stress-strain alignment behaves like that in non-reacting turbulence in the preheat zone of the flame. In all the cases investigated, it has been observed that the stress-strain alignment is affected by the increased kinematic viscosity and dilatation effects on the burned gas side of the flame. Sensitivity analysis for the choice of the filter width suggests that the stress-strain alignment is not significantly affected in the case of low turbulence intensity flames, but in the case of high turbulence intensity flames the alignment is altered by the change in filter width. The findings in this work suggest that the modelling of SGS stress should explicitly account for the effects of heat release and relative alignment of the SGS stress with the resolved part of the strain rate in LES of turbulent reacting flows. As this paper presents the very first analysis of the relative alignment between the SGS stress and the resolved strain rate for premixed flames, these statistics have been discussed for unity Lewis number conditions in the absence of differential diffusion effects of heat and mass. This is representative of usual hydrocarbon-air mixtures (e.g. stoichiometric methane-air mixture) which do not show significant departures from unity Lewis (*Le*) number. The investigation of non-unity Lewis number flames is beyond the scope of current work and will be addressed in future studies. However, the effects of heat release rate in turbulent premixed flames strengthen with decreasing Lewis number and thus it is expected that the stress-strain alignment for flames with *Le* ≪ 1 are expected to be qualitatively similar to that observed for unity Lewis number flames with smaller turbulence intensity^4,6^.

## Methods

A DNS database of freely propagating statistically planar turbulent premixed flames has been considered for this analysis. The simulations have been conducted using a three dimensional compressible code, SENGA^14^, which employs high-order finite-difference (10^th^ order for internal points and gradually decreasing to 2^nd^ order at the non-periodic boundaries) and Runge-Kutta (3^rd^ order explicit) schemes for spatial differentiation and time advancement, respectively. The simulation domain is discretised using a uniform Cartesian grid of dimension 800 × 400 × 400, which ensures 10 grid points across *δ*_*th*_. This grid resolution also ensures that the Kolmogorov scale is at least 1.5 times of the grid spacing at all the turbulence intensities considered in this work. A single-step chemical mechanism has been used for the purpose of computational economy and also due to the fact that the fundamental turbulence-chemistry interaction investigated in this work is unaffected by the chemical mechanism. A modified form of Lundgren’s forcing developed by Klein *et al*.^15^ is used to maintain the specified values of turbulence intensities and integral length scales upstream of the flame. The heat release parameter *τ* = (*T*_*ad*_ − *T*_*R*_)/*T*_*R*_ = 4.5, Zeldovich number ${\beta }_{z}={T}_{ac}({T}_{ad}-{T}_{R})/{T}_{ad}^{2}=6.0$, (where *T*_*ac*_ is the activation temperature) and the length scale ratio *l*_*t*_/*δ*_*z*_ = 5.3 are kept unaltered for all the cases considered in this work. In all cases the boundaries in the *x* direction are taken to be turbulent inflow and partially non-reflecting outflow, respectively and are specified using the Navier-Stokes characteristic boundary condition technique, while the boundaries in the transverse direction are treated as periodic. Further details about the DNS dataset can be found in^16^. In this analysis, the DNS data has been explicitly filtered using a Gaussian filter kernel defined as *G*(***r***) = (6/*π*Δ^2^)^3/2^ exp(−6***r***⋅***r***/Δ^2^). The results for two different filter widths 1.4*δ*_*z*_, and 3.5*δ*_*z*_ have been reported in this work.
